# Trends in Aeroallergen IgE Sensitization in Jordan: A Decade‐Long Observational Study

**DOI:** 10.1002/iid3.70533

**Published:** 2026-10-06

**Authors:** Jumana Abu‐Kwaik, Aymen Rawashdeh, Badia Saddedin, Mohammad Ghunaim, Thaer Hasan, Amid Abdelnour, Issa Abu‐Dayyeh

**Affiliations:** ^1^ Department of Research and Development Biolab Diagnostic Laboratories Amman Jordan; ^2^ Faculty of Science/Statistics Department Yarmouk University Irbid Jordan; ^3^ Department of Diagnostic Immunology Biolab Diagnostic Laboratories Amman Jordan

**Keywords:** allergen, allergic disease, cohort study, COVID‐19, epidemiology, Middle East and North Africa

## Abstract

**Background:**

Inhalant allergies from airborne allergens, such as pollen, dust mites, and animal dander, substantially contribute to allergic respiratory diseases globally. Sensitization to aeroallergens is influenced by environmental, urban, and climatic factors and demographic characteristics, including age and sex. However, long‐term data from large non–disease‐specific patient cohorts in Jordan are unavailable, particularly those covering the COVID‐19 pandemic years.

**Objective:**

To characterize prevalence and patterns of aeroallergen‐specific IgE (s‐IgE) sensitization in Jordan over a 10‐year period (2012–2022), evaluating associations with age, sex, and temporal trends.

**Methods:**

We retrospectively analyzed 4675 serum samples from patients tested at Biolab Diagnostic Laboratories. Aeroallergen s‐IgE was measured using the CE‐certified EUROLINE immunoblot panel covering 29 inhalant allergens. Samples positive for cross‐reactive carbohydrate determinants (2.4%) or repeat tests (3.8%) were excluded. Multivariable logistic regression assessed associations of sensitization with sex, age group (<20, 20–40, >40 years), and sample collection period (2012–2015, 2016–2019, 2020–2022).

**Results:**

Overall, 45.5% were sensitized to ≥1 aeroallergen. Males had higher odds of sensitization than females (OR = 1.35, *p* < 0.0001). Sensitization was most prevalent in the 20–40‐year age group. Temporal analysis showed an increase in overall sensitization from 40% in 2012–2015 to 50.6% in 2020–2022. The most common sensitizers were predominantly pollens (olive pollen being the single most prevalent at 21.9%), along with cat dander (17.5%) and dust mites (~12% combined). A mixed pattern of increased sensitization to both outdoor and indoor aeroallergens was observed during the pandemic years.

**Conclusions:**

Aeroallergen sensitization in Jordan shows significant sex‐, age‐, and time‐related patterns, with an overall increasing trend over the past decade, including during the COVID‐19 pandemic. These findings provide a robust epidemiologic baseline to inform public health strategies, allergen mitigation, and clinical management in urban and regional contexts.

## 1 Introduction

Inhalant allergies, caused by airborne allergens such as pollen, dust mites, and animal dander, are a growing public health concern worldwide [1]. They play a significant role in the rising global burden of allergic respiratory diseases, especially allergic rhinitis (AR) and asthma, which affect more than 400 million and 300 million individuals worldwide, respectively [2]. A key factor in the onset and progression of these conditions is sensitization to inhalant allergens. This process begins when the immune system produces allergen‐specific immunoglobulin E (s‐IgE) antibodies upon initial exposure. These antibodies bind to mast cell receptors, priming them for activation and subsequent release of inflammatory mediators, triggering allergic symptoms [3].

Climate change and urbanization significantly influence the prevalence and distribution of aeroallergens, shaping allergic disease patterns worldwide. Rising temperatures and increased carbon dioxide levels in the atmosphere have been shown to extend pollen seasons, boost allergen production, and expand the geographical range of allergenic plants, leading to higher sensitization rates and more severe allergic conditions [4]. In Jordan, rapid urbanization, especially in the capital Amman, has further complicated these challenges [5]. Expanding infrastructure increases air pollution, which can worsen respiratory conditions and enhance the allergenicity of airborne particles [6]. These environmental and urban changes not only increase allergen exposure but also contribute to heterogeneous and evolving sensitization profiles, underscoring the need for objective and standardized biomarkers to accurately characterize allergic sensitization at the population level. With allergic diseases on the rise locally, regionally, and globally [7], reliable diagnostic tools are essential for identifying sensitized individuals. Enzyme‐based immunoassays, including enzyme allergosorbent tests (EAST), are widely used for the detection of s‐IgE antibodies in serum. This minimally invasive approach requires only a blood sample and serves as a valuable adjunctive tool for the characterization of allergic sensitization to both food and inhalant allergens [8, 9]. As environmental and urban factors continue to drive changes in aeroallergen exposure, accessible and standardized diagnostic tools, such as EAST, are expected to play a more and more vital role in improving disease management and guiding public health interventions.

Jordan's geographic and climatic diversity makes it an important setting for studying aeroallergen prevalence and its impact on public health. The country lies at the intersection of Mediterranean and arid desert climates, which influences the distribution and concentration of airborne allergens [10]. In Mediterranean regions, allergenic plants like olive trees, cypress, and grasses contribute to seasonal pollen exposure [11], while arid and semi‐arid areas generate high levels of dust and sand, both of which exacerbate respiratory conditions [12]. Despite these factors, current research on aeroallergen prevalence in Jordan and the Middle East remains limited and fragmented [13]. A major challenge is the lack of long‐term observational studies, which makes it difficult to track sensitization trends over time. Moreover, there is little or no data from the region covering the COVID‐19 pandemic years, during which unprecedented non‐pharmaceutical interventions (NPIs) were imposed by governments to control disease spread [14].

In this manuscript, we retrospectively analyzed aeroallergen s‐IgE data from over 4600 patients in Jordan collected over a decade (2012–2022), and evaluated the influence of sex, age, and time period on sensitization patterns. This study addresses a critical knowledge gap by providing a long‐term large‐scale epidemiological analysis of IgE sensitization to a broad range of aeroallergens in Jordan. Furthermore, it provides novel insights into previously understudied trends in sensitization during the COVID‐19 pandemic years (2020–2022), a period marked by strict NPIs implemented locally and regionally.

## 2 Materials and Methods

### 2.1 Aeroallergen s‐IgE Test

The CE‐certified, IVD‐approved EUROLINE immunoglobulin test strip Inhalation “Turkey 1” (Catalog #DP 3119‐1601‐11 E; Euroimmun, Lübeck, Germany) was used to semi‐quantitatively measure aeroallergen s‐IgE levels. Strips were processed with the EUROBlotMaster (Euroimmun) following the manufacturer's guidelines, with each strip coated with 29 aeroallergens (grass mix, early [gs12]; grass mix, late [gs15]; grain pollen mix [gs21]; cultivated rye [g12]; tree mix, early [ts23]; tree mix, late [ts24]; olive [t9]; mulberry tree [t70]; flower mix 3 [ws18]; weed mix 1 [ws19]; weed mix 3 [ws20]; dermatoph pteronyssinus [d1]; dermatoph farinae [d2]; cockroach, German [i6]; feather mix 1 [es2]; cage bird mix 2 [es172]; cat [e1]; dog [e2]; horse [e3]; cow [e4]; goat [e80]; sheep [e81]; hamster [e84]; exotic mould mix 1 [ms11]; exotic mould mix 2 [ms12]; penicillin notatum [m1]; cladosporium herbarum [m2]; aspergillus fumigatus [m3]; alternaria alternata [m6]), a cross‐reactive carbohydrate determinant (CCD), and an indicator band. Strips were moistened, then incubated with 400 μL of undiluted fresh patient serum to capture any present s‐IgE antibodies. Bound s‐IgE antibodies were then detected using an enzyme‐linked anti‐human IgE, producing a colorimetric reaction, as previously described [8]. EUROLineScan software (Euroimmun) was used to scan and analyze the strips for enzyme allergosorbent test (EAST) class classification. Negative results, indicating the absence of detectable s‐IgE, were labeled as class 0 (< 0.35 kU/L), while positive results ranged from EAST class 1 to 6, with class 1 indicating the lowest (0.35 ≤ s‐IgE < 0.7 kU/L) and class 6 the highest (≥ 100.0 kU/L) s‐IgE levels.

### 2.2 Patient Data and Inclusion/Exclusion Criteria

Patient‐related sex, age, geographic location, sample time period, and s‐IgE test data were retrospectively collected from records of individuals who had previously undergone EAST testing at Biolab Laboratory. Data were accessed through the laboratory's Information Management System (LIMS), with participant anonymity maintained. The study included individuals of any age and sex who underwent aeroallergen s‐IgE testing at Biolab, either by physician referral (64%) or personal request (36%), from January 2012 to December 2022. The 4675 samples analyzed in this study were obtained from 10 of the 12 Jordanian governorates, predominantly from the capital, Amman (93.5%), followed by Zarqa (2.0%), Balqa (1.4%), Aqaba (1.0%), Irbid (0.8%), Tafila (0.5%), Jerash (0.3%), Madaba (0.2%), Ma'an (0.2%), and Karak (0.1%). Eighty‐eight percent of samples were collected from walk‐in patients attending 1 of 22 Biolab branches operating across major urban centers in Jordan at the time of collection, and serving a diverse population [15, 16, 17, 18, 19]. The remaining 12% originated from referral laboratories requesting s‐IgE testing through Biolab's immunology department. Repeat tests (*N* = 179) and those positive for cross‐reactive carbohydrate determinants (CCD) (*N* = 113) were excluded from the study to avoid overrepresentation and cross‐reactivity bias, respectively.

### 2.3 Graphs and Statistical Analyses

Multivariable logistic regression was used to assess the association between aeroallergen sensitization (dependent variable, defined as EAST class ≥ 1) and independent variables including gender, age group (< 20, 20–40, > 40 years), and sample collection period (2012–2015, 2016–2019, 2020–2022). An additional multivariable logistic regression model was performed to assess the association between encounter type (physician referral vs. personal request) and overall aeroallergen sensitization, while adjusting for the same covariates. Multivariable regression models were used to estimate adjusted associations while controlling for potential confounders. Results were reported as odds ratios (ORs) with *p*‐values and corresponding 95% CIs.

For supplementary analyses, comparisons were performed on aggregated sensitization prevalence values calculated separately for each allergen. In Supporting Information S1: Figure 1, male and female sensitization prevalences were paired by allergen, and differences were assessed using the Wilcoxon signed‐rank test. In Supporting Information S1: Figures 2 and 3, allergen‐specific sensitization prevalences across age groups and time periods were compared using the Friedman test, treating allergen as the repeating factor.

Missing values (<0.1%) were excluded from analysis. Statistical significance was set at *p* < 0.05. All analyses and graphs were generated using R (version 4.4.3) with the ggplot2 package.

### 2.4 Oversight

The institutional review board at Biolab (IRB‐Bio/4‐2022/01) approved this study with a waiver of informed consent due to the retrospective and anonymized nature of the study.

## 3 Results

### 3.1 Characterization of the Total Study Population

Of the 4675 individuals included in the study, 47.4% were male (age range: 6 days to 94 years; mean age: 26.5 years), and 52.6% were female (age range: 4 days to 95 years; mean age: 30.8 years) (Figure 1A). Age group distribution was as follows: 37.5% were <20 years, 35.3% were 20–40 years, and 27.2% were >40 years (Figure 1B). Twenty‐three percent of tests were conducted between 2012 and 2015 (time period 1, TP1), 46% between 2016 and 2019 (TP2), and 31% between 2020 and 2022 (TP3) (Figure 1C).

**Figure 1:**
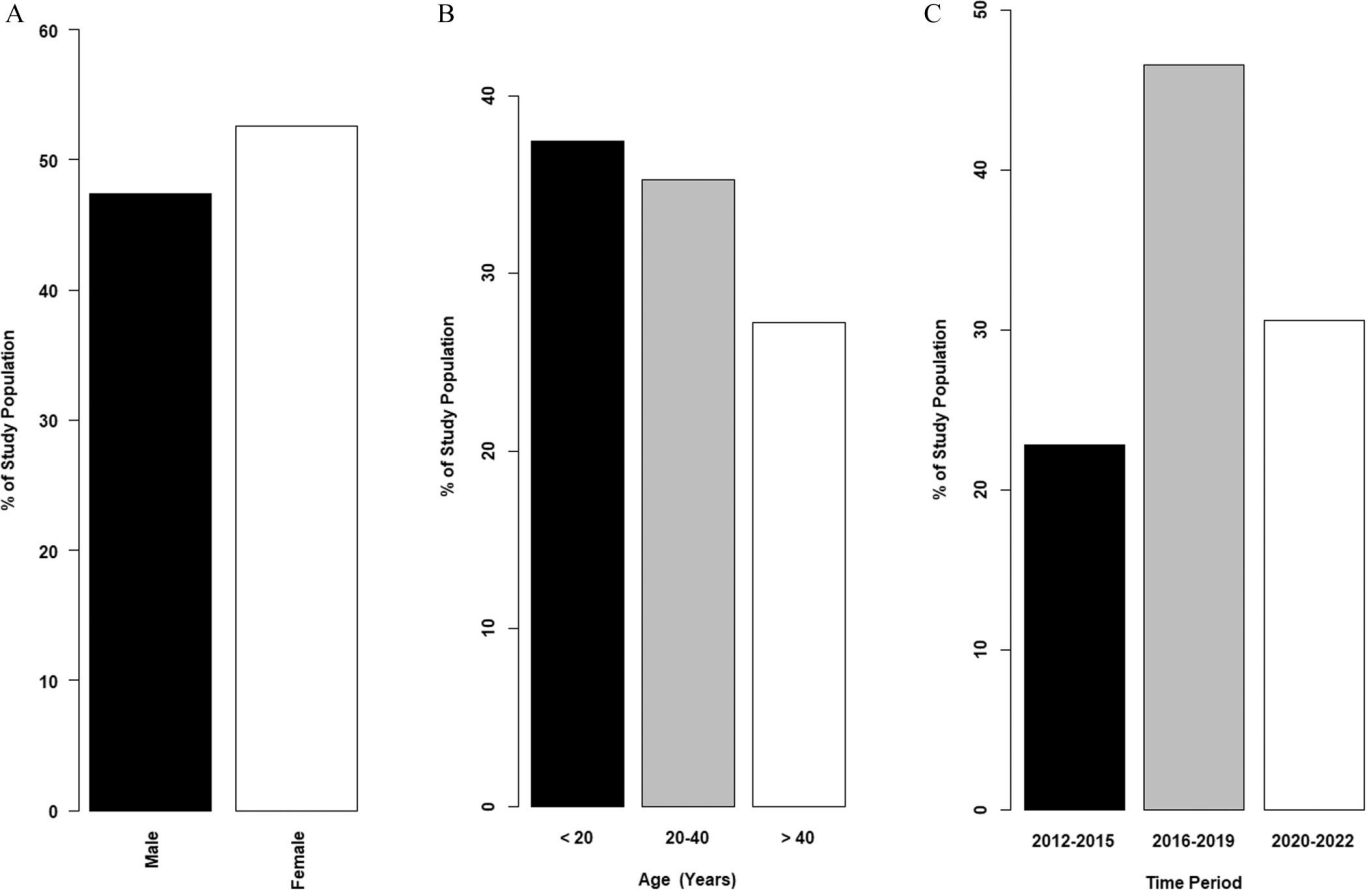
Characteristics of the total study population. (A) Bar graph illustrating the distribution of sexes across the study population. (B) Bar graph depicting the defined age group distribution of the study population. (C) Bar graph showing the percentage of the study population across the defined time periods.

### 3.2 Characterization of the s‐IgE‐Sensitized Population

Overall, 45.5% were sensitized to one or more aeroallergens on the immunoblot panel (Figure 2A). Logistic regression showed that males had higher odds of s‐IgE positivity than females (odds ratio [OR] = 1.35, 95% CI = 1.20–1.52, *p* < 0.0001; Figure 2B). The higher prevalence in males was consistent across the 12 most common allergens (Supporting Information S1: Figure 1). The 20–40‐year age group had the highest s‐IgE prevalence (51.8%). Compared with this group, the <20‐year group had lower odds of s‐IgE positivity (OR = 0.69, 95% CI = 0.60–0.79, *p* < 0.0001), as did the >40‐year group (OR = 0.61, 95% CI = 0.52–0.70, *p* < 0.001) (Figure 2C). The higher prevalence in the 20–40‐year group was also consistent across the 12 most common allergens (Supporting Information S1: Figure 2). By time period, sensitization prevalence increased from 40% in TP1 to 44.8% in TP2 and 50.6% in TP3. Logistic regression showed lower odds of s‐IgE positivity in TP1 (OR = 0.65, 95% CI = 0.55–0.77, *p* < 0.0001) and TP2 (OR = 0.79, 95% CI = 0.69–0.90, *p* < 0.0001) compared with TP3 (Figure 2D). This increasing trend in prevalence over the 10‐year period was consistent across the majority of the 12 most common allergens (Supporting Information S1: Figure 3). Further analysis indicated that pollens, molds, and dander were the major aeroallergen groups with significantly increased sensitization during TP3 (Supporting Information S1: Table 1). Logistic regression analyses of individual allergens within each group confirmed that most allergens contributed to this increase, with the exception of mulberry tree pollen, *Cladosporium herbarum*, *Penicillium notatum* molds, goat dander, cage birds, and feather mix (Supporting Information S1: Table 2).

**Figure 2:**
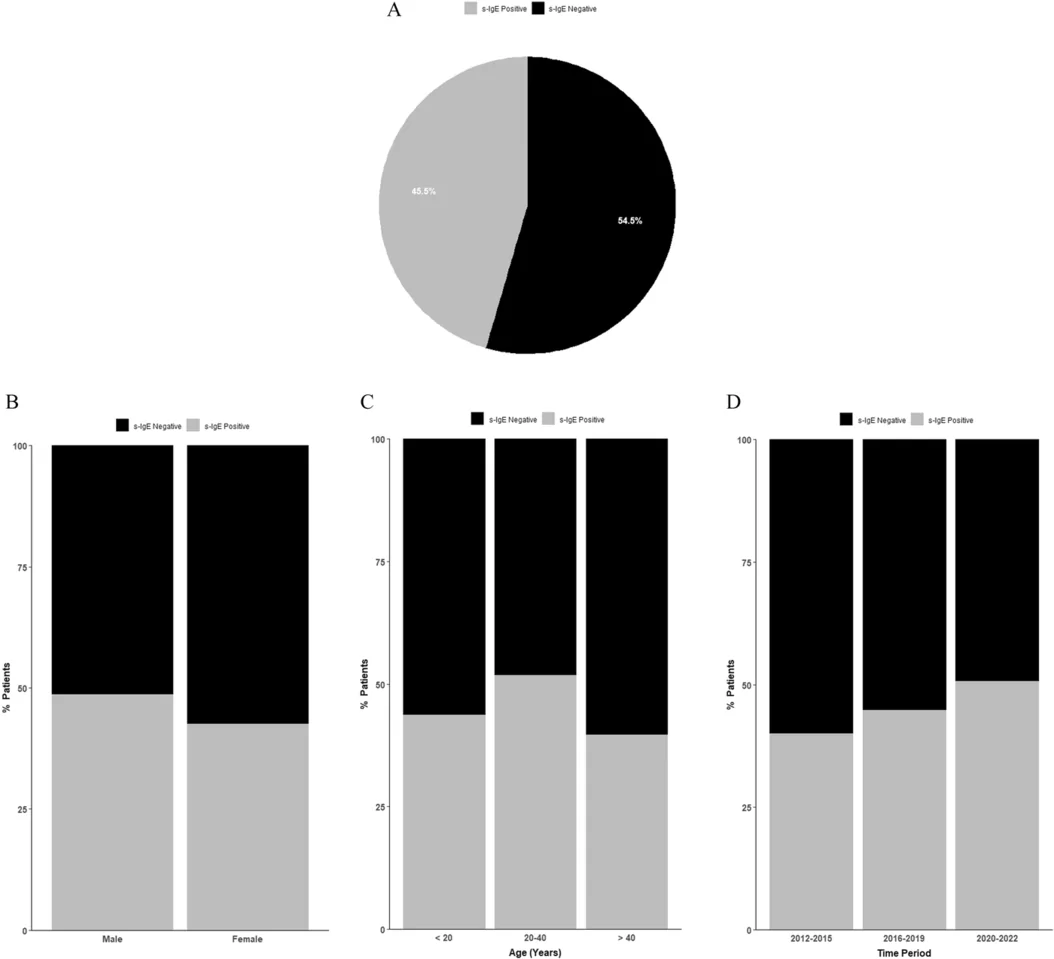
Characteristics of the s‐IgE‐sensitized population. (A) Pie chart showing the percentage of the IgE‐sensitized patients (gray) relative to the total study population. (B) Bar graph representation of contingency table data. Each bar represents one gender and is divided based on the percentage presence or absence of aeroallergen s‐IgE antibodies in patients' sera. Odds ratio (ref: Female) = 1.35, *p* < 0.0001, 95% CI = 1.20–1.52. (C) Bar graph representation of contingency table data. Each bar represents one age group and is divided based on the percentage presence or absence of aeroallergen s‐IgE antibodies in patients' sera. Odds ratio for <20 (ref: 20–40) = 0.69, *p* < 0.0001, CI = 0.60–0.79, odds ratio for >40 (ref: 20–40) = 0.61, *p* < 0.0001, 95% CI = 0.52–0.70. (D) Bar graph representation of contingency table data. Each bar represents one time period and is divided based on the percentage presence or absence of aeroallergen s‐IgE antibodies in patients' sera. Odds ratio for 2012–2015 (ref: 2020–2022) = 0.65, *p* < 0.0001, CI = 0.55–0.77, odds ratio 2016–2019 (ref: 2020–2022) = 0.79, *p* < 0.0001, CI = 0.69–0.9, odds ratio 2012–2015 (ref: 2016–2019) = 0.83, *p* = 0.014, CI = 0.71–0.96.

### 3.3 Prevalence of s‐IgE to Individual Aeroallergens

Among s‐IgE positive individuals, olive tree pollen had the highest prevalence (21.9%, 95% CI = 20.7%–23.1%), followed by late tree pollens (19.1%, 95% CI = 17.9%–20.2%), and cat dander (17.5%, 95% CI = 16.4%–18.6%). Late and early grass pollens ranked fourth and fifth (16.3%, 95% CI = 15.2%–17.4% and 14.5%, 95% CI = 13.5%–15.6%), grain pollens sixth and seventh (14.4%, 95% CI = 13.4%–15.4% and 11%, 95% CI = 10.1%–12%), and herb pollen eighth (10.4%, 95% CI = 9.6%–11.4%). Dust mite species, *Dermatophagoides pteronyssinus* and *D. farinae*, ranked eleventh and twelfth, respectively (*D. pteronyssinus*: 6.4% (95% CI = 5.7%–7.1%) and *D. farinae*: 5.8% (95% CI = 5.2%–6.5%)). The lowest prevalence was observed for cage birds (0.5%, 95% CI = 0.3%–0.7%), feather mix (0.7%, 95% CI = 0.5%–1%), and *Cladosporium herbarum* (0.8%, 95% CI = 0.6%–1.1%) (Figure 3). Analysis of EAST class distribution showed that the 12 most common allergens (Figure 3) had the highest fractions of medium and high s‐IgE titers. However, the dander group, comprising hamster, dog, cow, horse, goat, and sheep allergens, exhibited high fractions of medium and high titers despite their relatively low prevalence in the population (Supporting Information S1: Figure 4).

**Figure 3:**
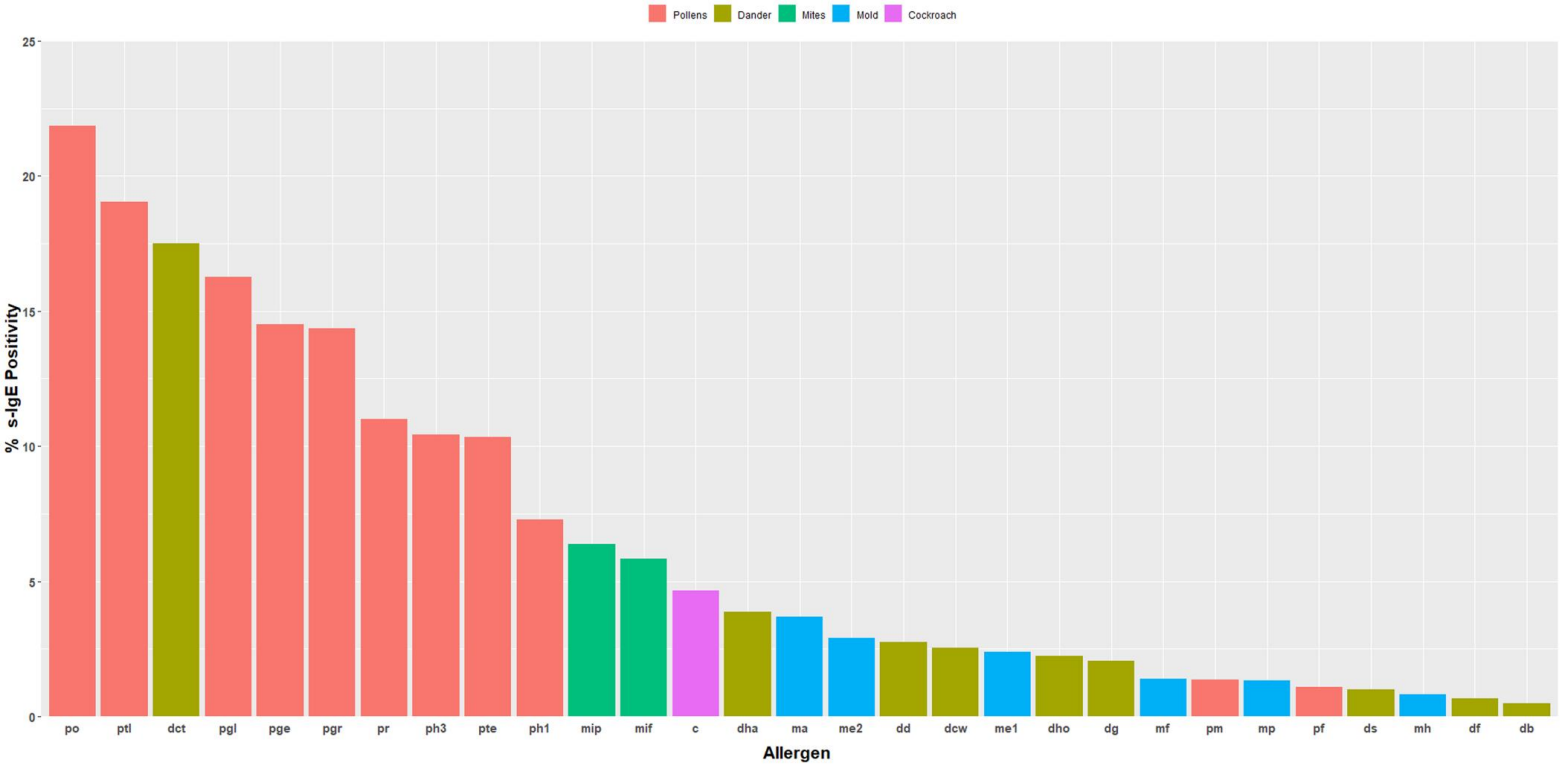
Prevalence of s‐IgE for individual and categorized aeroallergens in the sensitized population. Bar graph representing the prevalence (%) of s‐IgE sensitization for each allergen (abbreviated), ranked from most to least prevalent. The aeroallergens were also color‐coded based on the broader group they belong to in order to highlight any existing sensitization trends. Abbreviations: po = olive tree, ptl = tree pollens, late, pgl = grass pollens, late, pge = grass pollens, early, pgr = grain pollens, pr = rye, ph3 = herb pollen 3, pte = tree pollens, early, ph1 = herb pollen 1, pm = mulberry tree, pf = flower mix 3, dct = cat, dha = hamster, dd = dog, dcw = cow, dho = horse, dg = goat, ds = sheep, df = feather mix, db = cage birds, mip = D. pteronyssinus, mif = D. farinae, c = cockroach, ma = *Alternaria alternata*, me2 = exotic mold mix 2, me1 = exotic mold mix 1, mf = *Aspergillus fumigatus*, mp = *Penicillium notatum*, mh = *Cladosporium herbarum*. Aeroallergen groups: pollens (salmon color), dander (pistachio color), mites (forest green), mold (blue), and cockroach (pink).

## 4 Discussion

The published literature on inhalant allergy in Jordan is limited in number and temporally fragmented. These studies typically examine heterogeneous allergen panels in relatively small, disease‐specific cohorts [20, 21, 22, 23, 24, 25, 26]. This heterogeneity, together with limited sample sizes, restricts both the generalizability of findings and meaningful cross‐study comparisons. The present study addresses these limitations by leveraging a large laboratory database from a network covering multiple regions in Jordan, spanning 10 years. Aeroallergen sensitization was assessed using a standardized assay, allowing consistent assessment of sensitization patterns over time on a single testing platform. This approach provides a robust framework to inform public health planning and mitigation strategies for aeroallergen‐related conditions in Jordan and other settings with similar aeroallergen exposures.

Although the laboratory network serves multiple regions across Jordan [15], approximately 94% of samples in this study were collected from the capital city, Amman. This distribution reflects both the concentration of healthcare and diagnostic services in the capital and the fact that Amman is home to more than 40% of Jordan's population [27]. While the remaining samples originated from other governorates, the corresponding laboratory branches are also predominantly located in urban or semi‐urban population centers rather than in distinctly rural settings. Furthermore, the relatively small proportion of samples originating outside Amman limits the ability to make robust comparisons between geographic regions or levels of urbanization. We therefore consider the present dataset more appropriately representative of predominantly urban populations and do not attempt to infer an effect of urbanization on aeroallergen sensitization. Nevertheless, the predominance of samples from Amman provides valuable insight into aeroallergen sensitization patterns in a highly urbanized setting and highlights the need for future population‐based studies specifically designed to compare urban and rural populations across Jordan.

Patients with IgE antibodies to CCDs were excluded from the analysis, as such antibodies are frequently non‐specific and often lack clinical relevance [28]. In this cohort, CCD sensitization was detected in 2.4% of individuals, substantially lower than the >18% reported in previous studies [28, 29, 30]. This prevalence closely matches the 2.3% CCD positivity observed in our prior study using a similar immunoblot assay for food allergens [8], suggesting that differences in assay characteristics (including CCD cut‐off definitions, sensitivity, and specificity) and population background (genetic and demographic factors) may contribute to these observed differences. Additional contributing factors include differences in environmental aeroallergen exposure and the fact that our analysis is limited to inhalant allergens, whereas many published reports assess CCD across both food and inhalant panels.

This study represents the largest aeroallergen IgE sensitization cohort reported from Jordan to date, with a primary sampling focus on the highly urbanized capital, Amman. The cohort included a balanced distribution of males and females (Figure 1A), strengthening analyses of sex‐related differences in sensitization. Participants were stratified into three age groups (<20 years, 20–40 years, >40 years), reflecting broadly representative life‐stage patterns of daily activity and environmental exposures, while also ensuring sufficiently sized groups for robust logistic regression analyses (Figure 1B). Testing dates were additionally grouped into consecutive 3–4‐year intervals, including a dedicated period corresponding to the COVID‐19 pandemic, during which extensive non‐pharmaceutical interventions (NPIs) were implemented in Jordan (Figure 1C).

Importantly, participants in our study were not randomly selected from the general population but underwent aeroallergen s‐IgE testing in a real‐world diagnostic setting, primarily following clinical referral or individual testing requests. The cohort is therefore likely to be clinically enriched for individuals with suspected or perceived allergic symptoms and should not be considered representative of the general Jordanian population. Accordingly, the overall sensitization rate observed in this study (45.5%; Figure 2A) should not be interpreted as a population‐based estimate of aeroallergen sensitization in Jordan. Rather, it reflects sensitization patterns among individuals undergoing aeroallergen s‐IgE testing within a large laboratory‐based cohort.

Given the potential for referral patterns to influence the observed sensitization profile, we further examined whether encounter type was associated with sensitization. An additional multivariable logistic regression analysis incorporating encounter type (physician referral vs. personal request) showed no significant association between encounter type and overall aeroallergen sensitization. Furthermore, comparison of the distribution of s‐IgE sensitization classes between encounter types showed broadly consistent patterns across the individual allergens tested. These findings suggest that the principal sensitization patterns observed in this cohort were not substantially influenced by whether testing was initiated through physician referral or individual request.

Nevertheless, comparisons with published studies provide useful context for interpreting the magnitude and pattern of sensitization observed in our cohort. The overall sensitization rate in our study falls slightly above the range reported in several large population‐based studies from Europe (32%–42%) [31], although direct comparisons should be interpreted cautiously given differences in population selection and study methodology. Conversely, substantially higher rates have been reported in disease‐specific cohorts, such as patients with allergic rhinitis in China (84%) [32] and Egypt (75%) [33], consistent with the expected enrichment of sensitized individuals in clinically defined populations.

Consistent with patterns reported in large multi‐center general population cohorts [34], aeroallergen sensitization in our cohort (45.5%) was higher than food allergen sensitization (24%) reported in a prior study from our laboratory using a similar cohort and assay [8]. This difference is expected, as aeroallergens are ubiquitous and result in continuous low‐level exposure, whereas exposure to food allergens is more limited and sensitization may wane in adulthood.

Males were found to have higher odds of being sensitized to one or more aeroallergens than females (Figure 2B). This male predominance in IgE sensitization has been consistently reported in the literature [35, 36]. Multiple factors, including genetic, hormonal, lifestyle, and environmental influences, have been proposed to contribute to sex‐related differences in IgE sensitization. Whether this observed sex bias translates into clinically relevant differences in allergic disease expression warrants further investigation.

Individuals aged 20–40 years exhibited the highest rate of aeroallergen s‐IgE sensitization compared with those younger than 20 and older than 40. This pattern is consistent with several reports showing increased sensitization as individuals transition from childhood to adolescence to early adulthood, with a subsequent decline in older adults, potentially reflecting age‐related immunological changes, including immunosenescence [32, 37, 38].

Temporally, an increasing trend in aeroallergen sensitization was observed over the 10‐year study period, including during the COVID‐19 pandemic years (Figure 2D). Temporal upward trends in aeroallergen IgE sensitization have been documented in population‐based studies worldwide [39, 40]. Notably, no decrease in sensitization prevalence was observed during the pandemic years in Jordan, despite widespread mask use and the implementation of NPIs, including restrictions on outdoor mobility. However, because sensitization reflects cumulative immune responses to allergen exposure and our study compared different individuals tested across successive time periods rather than longitudinally following the same individuals, the observed temporal increase cannot be directly attributed to pandemic‐related changes in allergen exposure or behavior. The pandemic period should therefore be regarded as a temporal context in which the observed sensitization patterns occurred rather than as evidence of a causal effect of COVID‐19‐related restrictions on sensitization.

During the COVID‐19 pandemic, we observed a mixed pattern of increased sensitization to both outdoor and indoor aeroallergens. Pollen and certain indoor allergens, including some types of animal dander and molds, increased, whereas other indoor allergens, such as dust mites and cockroaches, remained relatively stable (Supporting Information S1: Figure 3). A more pronounced indoor‐allergen‐focused increase in sensitization was reported in a recent Chinese study [41], likely reflecting the strict home quarantine policies implemented there, while a mixed outdoor/indoor pattern was reported by a recent study from Turkey [42]. Although these findings provide an interesting temporal context, our observational data cannot establish that pandemic‐related restrictions or changes in behavior caused the observed changes in sensitization. Changes in healthcare‐seeking behavior, testing patterns, environmental exposures, and other unmeasured factors during the pandemic may also have contributed to the observed temporal differences. The increase observed during 2020–2022 should therefore be interpreted as an epidemiological temporal trend rather than as evidence that pandemic‐related behavioral changes directly altered aeroallergen sensitization.

Analysis of the aeroallergen sensitization panel revealed that the top 12 allergens, out of a total of 29, represented the higher‐prevalence subset of sensitizers (Figure 3) and were characterized by medium to high IgE titers (Supporting Information S1: Figure 4), suggesting potential clinical relevance and supporting their prioritization in the present analysis. These top allergens were predominantly pollens, with olive pollen emerging as the most common sensitizer, consistent with the widespread presence of olive trees in Jordan [43] and previous reports identifying olive as a major contributor to allergic sensitization in the country and the region [20, 21, 23, 25, 26, 44, 45].

Cat dander also featured prominently, in line with prior Jordanian studies [20, 22, 23]. Notably, sensitization may occur despite relatively low indoor pet ownership, likely reflecting the ubiquitous presence of outdoor and stray cats and the capacity of cat allergens to be transported via clothing and environmental dust into homes and shared spaces [20]. In addition, dust mites ‐long recognized contributors to allergic sensitization‐ were present among the top 12, consistent with findings from national [20, 21, 22, 23, 24, 25, 26], regional [46, 47], and international studies [48]. Importantly, supplementary analyses stratified by gender, age group, and time period (Supporting Information S1: Figures 1–3) showed that the overall ranking of these top allergens remained largely unchanged, underscoring the robustness of the main findings illustrated in Figure 3.

An additional noteworthy observation was that several allergens within the dander group, despite having relatively low sensitization prevalence, demonstrated a disproportionately high distribution of intermediate to high IgE classes (Supporting Information S1: Figure 4). One plausible explanation for this pattern is immunologic cross‐reactivity with cat allergens, which were among the most prevalent sensitizers in this cohort. Mammalian dander allergens are known to share homologous protein families, particularly serum albumins and lipocalins, that can elicit cross‐reactive IgE responses across species. For example, cat serum albumin (Fel d 2) shows structural similarity and documented cross‐reactivity with albumins from dog (Can f 3), horse (Equ c 3), cow (Bos d 6), and other mammalian sources, while lipocalin allergens such as Fel d 4 exhibit homology with lipocalins from multiple domestic and farm animals [49]. Such cross‐sensitization may lead to measurable IgE responses to less frequently encountered animal dander, even in the absence of significant direct exposure.

Based on the sensitization patterns observed in Figure 3, several practical mitigation strategies can be considered. The predominance of pollen sensitization—particularly to olive pollen—supports allergen‐aware urban landscaping, including limiting the planting of highly allergenic olive trees in densely populated areas and promoting the use of lower‐allergenic plant species, alongside public access to seasonal pollen forecasts and preventive measures during peak seasons. For cat dander sensitization, structured community animal‐management programs and targeted public‐health education may help reduce overall environmental exposure. Standard environmental control measures remain relevant for dust mite sensitization, including humidity control and allergen‐impermeable bedding. Finally, improving access to allergen immunotherapy through standardized national protocols aligned with prevalent allergens and enhanced affordability and availability may offer an additional strategy for patients with persistent, clinically significant disease.

This study has limitations. First, it relies on real‐world data, which are inherently constrained by their retrospective nature and reliance on electronic health records rather than prospective designs tailored to estimating specific epidemiologic parameters or establishing causal relationships. Second, detailed clinical and behavioral information of the participants was not available, limiting the ability to correlate sensitization patterns with symptom severity, environmental exposures, or lifestyle factors. Finally, temporal changes in testing practices, healthcare utilization, and access to diagnostic services over the last decade may have influenced the observed trends in sensitization.

Despite these limitations, the study has notable strengths. It utilizes a large laboratory database with national coverage over a 10‐year period, providing a comprehensive view of allergen sensitization in Jordan. Furthermore, the use of a single, reliable assay throughout the study period ensures consistency and comparability of results. To our knowledge, this represents the most detailed analysis of aeroallergen sensitization in Jordan to date, offering valuable national epidemiological and clinical insights into dominant aeroallergens, temporal trends in sensitization, and their associations with age, sex, and IgE titers. These findings provide a strong foundation and serve as a complementary resource for future nationwide population‐based surveys aimed at further refining public health strategies and clinical management.

## Author Contributions

**Jumana Abu‐Kwaik:** data curation, methodology, formal analysis, validation, writing – original draft. **Aymen Rawashdeh:** methodology, formal analysis, writing – review and editing, data curation, visualization, validation. **Badia Saddedin:** writing – original draft. **Mohammad Ghunaim:** software, data curation. **Thaer Hasan:** software, data curation. **Amid Abdelnour:** funding acquisition, resources. **Issa Abu‐Dayyeh:** conceptualization, methodology, investigation, formal analysis, supervision, funding acquisition, project administration, resources, writing – original draft, writing – review and editing, validation.

## Ethics Statement

The institutional review boards at Biolab (ethics approval number: IRB‐Bio/4‐2022/01) approved this retrospective study with a waiver of informed consent.

## Conflicts of Interest

The authors declare no conflicts of interest.

## Declaration of AI Use

ChatGPT version 5 was used for grammar verification and phrasing refinement. The authors thoroughly reviewed and edited the text, taking full responsibility for its accuracy and quality.

## Supporting information


Supporting File

## Data Availability

The dataset of this study is a property of Biolab Diagnostic Laboratories that was provided to the researchers for scientific research purposes through a restricted‐access agreement that prevents sharing the dataset with a third party or publicly. The data are available upon reasonable request and under restricted access for preservation of confidentiality of patient data. Access can be obtained through a direct application for data access to Dr. Issa Abu‐Dayyeh, head of research and development department at Biolab Diagnostic Laboratories, E‐mail: i.abudayyeh@biolab.jo. The raw data are protected and are not available due to data privacy laws. Data were available to authors through .csv files (no links/accession codes were available to authors).
