# Supplementary material for: Trends in Aeroallergen IgE Sensitization in Jordan: A Decade‐Long Observational Study
Source: Immun Inflamm Dis. 2026 Oct 6;14(10):e70533. doi: 10.1002/iid3.70533 (PMC13640519; doi:10.1002/iid3.70533)
Supplement: Supplementary file 1 [file IID3-14-e70533-s001.doc]

**Supplementary Figure 1.** Prevalence of s-IgE across aeroallergens by gender. Bar graphs illustrating the prevalence (%) of s-IgE sensitization for allergens in males and females, displayed separately. Abbreviations: po=olive tree, ptl=tree pollens, late, pgl=grass pollens, late, pge=grass pollens, early, pgr=grain pollens, pr=rye, ph3=herb pollen 3, pte=tree pollens, early, ph1=herb pollen 1, pm=mulberry tree, pf=flower mix 3, dct=cat, dha=hamster, dd=dog, dcw=cow, dho=horse, dg=goat, ds=sheep, df=feather mix, db=cage birds, mip=D. Pteronyssinus, mif=D. Farinae, c=cockroach, ma=Alternaria alternata, me2=exotic mold mix 2, me1=exotic mold mix 1, mf=aspergillus fumigatus, mp=Penicillium notatum, mh=Cladosporium herbarum. Aeroallergen groups: pollens (salmon color), dander (pistachio color), mites (forest green), mold (blue), and cockroach (pink).

**Supplementary Figure 2.** Prevalence of s-IgE across aeroallergens by age group. Bar graphs illustrating the prevalence (%) of s-IgE sensitization for allergens in three defined age groups, each displayed separately. Abbreviations: po=olive tree, ptl=tree pollens, late, pgl=grass pollens, late, pge=grass pollens, early, pgr=grain pollens, pr=rye, ph3=herb pollen 3, pte=tree pollens, early, ph1=herb pollen 1, pm=mulberry tree, pf=flower mix 3, dct=cat, dha=hamster, dd=dog, dcw=cow, dho=horse, dg=goat, ds=sheep, df=feather mix, db=cage birds, mip=D. Pteronyssinus, mif=D. Farinae, c=cockroach, ma=Alternaria alternata, me2=exotic mold mix 2, me1=exotic mold mix 1, mf=aspergillus fumigatus, mp=Penicillium notatum, mh=Cladosporium herbarum. Aeroallergen groups: pollens (salmon color), dander (pistachio color), mites (forest green), mold (blue), and cockroach (pink).

**Supplementary Figure 3.** Prevalence of s-IgE across aeroallergens by time period. Bar graphs illustrating the prevalence (%) of s-IgE sensitization for allergens in three defined time periods, each displayed separately. Abbreviations: po=olive tree, ptl=tree pollens, late, pgl=grass pollens, late, pge=grass pollens, early, pgr=grain pollens, pr=rye, ph3=herb pollen 3, pte=tree pollens, early, ph1=herb pollen 1, pm=mulberry tree, pf=flower mix 3, dct=cat, dha=hamster, dd=dog, dcw=cow, dho=horse, dg=goat, ds=sheep, df=feather mix, db=cage birds, mip=D. Pteronyssinus, mif=D. Farinae, c=cockroach, ma=Alternaria alternata, me2=exotic mold mix 2, me1=exotic mold mix 1, mf=aspergillus fumigatus, mp=Penicillium notatum, mh=Cladosporium herbarum. Aeroallergen groups: pollens (salmon color), dander (pistachio color), mites (forest green), mold (blue), and cockroach (pink).

**Supplementary Table 1.** Associations between aeroallergen group sensitization and sex, age, and time period. Table summarizing the odds ratios, p-values, and corresponding 95% confidence intervals (lower and upper limits) for sensitization across grouped aeroallergens (pollens, mold, mites, dander, and cockroach). Comparisons were made according to sex (female vs. male), age groups (<20 years vs. 20–40 years, >40 years vs. 20–40 years), and time periods (TP1/TP3 and TP2/TP3). A p-value <0.05 was considered statistically significant. Abbreviations: TP=Time Period, TP1 (2012-2015), TP2 (2016-2019), TP3 (2020-2022).

|  | **Pollen** | | | |
| --- | --- | --- | --- | --- |
|  | Odds Ratio | p-value | Lower | Upper |
| Sex (Female/Male) | 0.780 | 0.000 | 0.688 | 0.884 |
| Age (<20 / [20-40]) | 0.713 | 0.000 | 0.617 | 0.823 |
| Age (>40 / [20-40]) | 0.624 | 0.000 | 0.532 | 0.731 |
| TP1/TP3 | 0.607 | 0.000 | 0.510 | 0.722 |
| TP2/TP3 | 0.822 | 0.006 | 0.715 | 0.946 |
|  |  |  |  |  |
|  | **Mold** | | | |
|  | Odds Ratio | p-value | Lower | Upper |
| Sex (Female/Male) | 0.737 | 0.007 | 0.589 | 0.920 |
| Age (<20 / [20-40]) | 1.135 | 0.317 | 0.886 | 1.456 |
| Age (>40 / [20-40]) | 0.644 | 0.005 | 0.471 | 0.874 |
| TP1/TP3 | 0.420 | 0.000 | 0.296 | 0.586 |
| TP2/TP3 | 0.727 | 0.008 | 0.574 | 0.922 |
|  |  |  |  |  |
|  | **Mites** | | | |
|  | Odds Ratio | p-value | Lower | Upper |
| Sex (Female/Male) | 0.720 | 0.004 | 0.575 | 0.900 |
| Age (<20 / [20-40]) | 0.666 | 0.002 | 0.511 | 0.866 |
| Age (>40 / [20-40]) | 0.800 | 0.111 | 0.606 | 1.051 |
| TP1/TP3 | 1.650 | 0.001 | 1.229 | 2.220 |
| TP2/TP3 | 1.107 | 0.466 | 0.845 | 1.457 |
|  |  |  |  |  |
|  | **Dander** | | | |
|  | Odds Ratio | p-value | Lower | Upper |
| Sex (Female/Male) | 0.820 | 0.005 | 0.713 | 0.942 |
| Age (<20 / [20-40]) | 0.985 | 0.849 | 0.843 | 1.151 |
| Age (>40 / [20-40]) | 0.521 | 0.000 | 0.431 | 0.628 |
| TP1/TP3 | 0.818 | 0.035 | 0.677 | 0.985 |
| TP2/TP3 | 0.740 | 0.000 | 0.633 | 0.867 |
|  |  |  |  |  |
|  | **Cockroach** | | | |
|  | Odds Ratio | p-value | Lower | Upper |
| Sex (Female/Male) | 0.620 | 0.001 | 0.468 | 0.818 |
| Age (<20 / [20-40]) | 0.507 | 0.000 | 0.356 | 0.717 |
| Age (>40 / [20-40]) | 1.040 | 0.808 | 0.755 | 1.430 |
| TP1/TP3 | 0.666 | 0.044 | 0.444 | 0.983 |
| TP2/TP3 | 0.876 | 0.396 | 0.646 | 1.193 |

**Supplementary Table 2. Individual allergen sensitization rate comparison between the different time periods. Table summarizing logistic regression and chi-square test results of each individual allergen tested over the different time periods (TP1/TP3 and TP2/TP3). A p-value <0.05 was considered statistically significant. Abbreviations: po=olive tree, ptl=tree pollens, late, pgl=grass pollens, late, pge=grass pollens, early, pgr=grain pollens, pr=rye, ph3=herb pollen 3, pte=tree pollens, early, ph1=herb pollen 1, pm=mulberry tree, pf=flower mix 3, dct=cat, dha=hamster, dd=dog, dcw=cow, dho=horse, dg=goat, ds=sheep, df=feather mix, db=cage birds, mip=D. Pteronyssinus, mif=D. Farinae, c=cockroach, ma=Alternaria alternata, me2=exotic mold mix 2, me1=exotic mold mix 2, mf=aspergillus fumigatus, mp=Penicillium notatum, mh=Cladosporium herbarum. Aeroallergen code highlighted in yellow indicates statistical significance in at least one time period comparison.**

|  | Logistic Regression Results | | | | chisq.test.Results |  |
| --- | --- | --- | --- | --- | --- | --- |
| Test | Odds.Ratio(TP1/TP3) | Odds.Ratio(TP2/TP3) | P.value_1 | P.value_2 | P.value_chisq.test | Group |
| pf | 0.224 | 0.622 | 0.006 | 0.107 | 0.008 | Pollens |
| pgr | 0.565 | 0.808 | 0.000 | 0.023 | 0.000 | Pollens |
| pge | 0.543 | 0.803 | 0.000 | 0.018 | 0.000 | Pollens |
| pgl | 0.395 | 0.777 | 0.000 | 0.004 | 0.000 | Pollens |
| ph1 | 0.882 | 1.305 | 0.457 | 0.044 | 0.012 | Pollens |
| ph3 | 1.616 | 1.448 | 0.000 | 0.002 | 0.001 | Pollens |
| pm | 1.306 | 1.617 | 0.484 | 0.131 | 0.229 | Pollens |
| po | 0.497 | 0.692 | 0.000 | 0.000 | 0.000 | Pollens |
| pr | 0.525 | 0.701 | 0.000 | 0.001 | 0.000 | Pollens |
| pte | 3.026 | 2.045 | 0.000 | 0.000 | 0.000 | Pollens |
| ptl | 0.494 | 0.600 | 0.000 | 0.000 | 0.000 | Pollens |
| ma | 0.380 | 0.960 | 0.000 | 0.810 | 0.000 | Mold |
| mf | 0.454 | 0.734 | 0.043 | 0.254 | 0.122 | Mold |
| mh | 1.343 | 1.170 | 0.512 | 0.692 | 0.799 | Mold |
| me2 | 0.459 | 0.741 | 0.004 | 0.113 | 0.014 | Mold |
| me1 | 0.259 | 0.548 | 0.000 | 0.003 | 0.000 | Mold |
| mp | 0.817 | 0.978 | 0.584 | 0.940 | 0.810 | Mold |
| mif | 0.913 | 1.116 | 0.613 | 0.449 | 0.445 | Mites |
| mip | 1.879 | 1.235 | 0.000 | 0.163 | 0.000 | Mites |
| db | 2.040 | 1.963 | 0.271 | 0.244 | 0.446 | Dander |
| dct | 0.714 | 0.770 | 0.002 | 0.003 | 0.001 | Dander |
| dcw | 0.290 | 0.515 | 0.000 | 0.001 | 0.000 | Dander |
| dd | 0.839 | 0.647 | 0.456 | 0.033 | 0.083 | Dander |
| df | 1.997 | 0.879 | 0.132 | 0.783 | 0.103 | Dander |
| dg | 0.189 | 1.053 | 0.001 | 0.814 | 0.000 | Dander |
| dha | 1.447 | 0.532 | 0.042 | 0.001 | 0.000 | Dander |
| dho | 0.663 | 0.602 | 0.128 | 0.023 | 0.055 | Dander |
| ds | 0.172 | 0.578 | 0.004 | 0.072 | 0.004 | Dander |
| c | 0.666 | 0.876 | 0.044 | 0.396 | 0.153 | Cockroach |

**Supplementary Figure 4.** S-IgE EAST class fraction distribution of total sensitized patients by aeroallergen. Stacked bar chart with each bar segmented to show the distribution of patients across three EAST class groups: low (1-2) in salmon color, medium (3-4) in green color and high (5-6) in blue color. Abbreviations: po=olive tree, ptl=tree pollens, late, pgl=grass pollens, late, pge=grass pollens, early, pgr=grain pollens, pr=rye, ph3=herb pollen 3, pte=tree pollens, early, ph1=herb pollen 1, pm=mulberry tree, pf=flower mix 3, dct=cat, dha=hamster, dd=dog, dcw=cow, dho=horse, dg=goat, ds=sheep, df=feather mix, db=cage birds, mip=D. Pteronyssinus, mif=D. Farinae, c=cockroach, ma=Alternaria alternata, me2=exotic mold mix 2, me1=exotic mold mix 1, mf=aspergillus fumigatus, mp=Penicillium notatum, mh=Cladosporium herbarum, EAST class groups: Low (1-2)=0.35≤sIgE<3.5 kU/L, medium (3-4)=3.5≤sIgE<50 kU/L, high (5-6)=sIgE≥50.0 kU/L.
